# Identification and Characterization of Novel Compounds Blocking Shiga Toxin Expression in *Escherichia coli* O157:H7

**DOI:** 10.3389/fmicb.2016.01930

**Published:** 2016-11-30

**Authors:** Alejandro Huerta-Uribe, Zoe R. Marjenberg, Nao Yamaguchi, Stephen Fitzgerald, James P. R. Connolly, Nuria Carpena, Hanna Uvell, Gillian Douce, Michael Elofsson, Olwyn Byron, Rudi Marquez, David L. Gally, Andrew J. Roe

**Affiliations:** ^1^Institute of Infection, Immunity and Inflammation, College of Medical, Veterinary and Life Sciences, University of GlasgowGlasgow, UK; ^2^Division of Immunity and Infection, The Roslin Institute and R(D)SVS, The University of EdinburghEdinburgh, UK; ^3^Laboratories for Chemical Biology Umeå, Department of Chemistry, Umeå UniversityUmeå, Sweden; ^4^School of Life Sciences, College of Medical, Veterinary and Life Sciences, University of GlasgowGlasgow, UK; ^5^Department of Chemistry, Xi’an Jiaotong-Liverpool UniversitySuzhou, China

**Keywords:** Shiga toxin, *E. coli*, RecA, phage, expression

## Abstract

Infections caused by Shiga toxin (Stx)-producing *E. coli* strains constitute a health problem, as they are problematic to treat. Stx production is a key virulence factor associated with the pathogenicity of enterohaemorrhagic *E. coli* (EHEC) and can result in the development of haemolytic uremic syndrome in infected patients. The genes encoding Stx are located on temperate lysogenic phages integrated into the bacterial chromosome and expression of the toxin is generally coupled to phage induction through the SOS response. We aimed to find new compounds capable of blocking expression of Stx type 2 (Stx2) as this subtype of Stx is more strongly associated with human disease. High-throughput screening of a small-molecule library identified a lead compound that reduced Stx2 expression in a dose-dependent manner. We show that the optimized compound interferes with the SOS response by directly affecting the activity and oligomerization of RecA, thus limiting phage activation and Stx2 expression. Our work suggests that RecA is highly susceptible to inhibition and that targeting this protein is a viable approach to limiting production of Stx2 by EHEC. This type of approach has the potential to limit production and transfer of other phage induced and transduced determinants.

## Introduction

Enterohemorrhagic *E. coli* (EHEC) are a group of Shiga toxin (Stx) producing pathogenic *E. coli* strains, which are associated with a broad spectrum of disease ranging from mild diarrhea to severe haemorrhagic colitis and haemolytic uremic syndrome (HUS) (Paton and Paton, 1998). The first recognition of an EHEC strain as a foodborne pathogen occurred in the US in 1982 during an investigation of customers from a fast-food restaurant chain who had bloody diarrhea and severe abdominal cramping with no fever (Riley et al., 1983). The *E. coli* serotype O157:H7, which had not previously been associated with human disease, was isolated from infected individuals and traced to contaminated hamburger meat (Riley et al., 1983). Shortly thereafter, *E. coli* O157:H7 was linked to the development of HUS (Karmali et al., 1983), a disease predominantly affecting children and carrying a 5-10% mortality rate. Since then, EHEC have been recognized as responsible for hundreds of food and waterborne outbreaks, with O157:H7 being the most prevalent serotype and the leading cause of HUS in Europe and the United States (Tarr et al., 2005; Lim et al., 2010).

The development of haemorrhagic colitis and HUS is dependent on the production of Stx, a family of related toxins that are essential for disease. Stx produced by EHEC strains are largely differentiated into two types that share 55% sequence homology (Fraser et al., 2004): Stx1, which differs from *Shigella dysenteriae* Stx by a single amino acid, and Stx2, which is structurally similar to Stx1 but antigenically distinct. Stx1 and Stx2 can be further classified into several subtypes based on the sequence-based relatedness of the proteins. These comprise three Stx1 subtypes (1a, 1c, 1d) and seven Stx2 subtypes (2a, 2b, 2c, 2d, 2e, 2f, and 2g).

Shiga toxin are AB_5_ toxins, which bind via the B subunits to the globotriaoslyceramide (Gb3) receptor, expressed on the surface of vascular endothelial cells as well as Paneth cells in the intestinal mucosa. B subunit binding leads to clathrin-dependent endocytosis of the A-B subunits. Following endosomal processing and trafficking to the golgi, the internalized A subunit cleaves the 28S ribosomal RNA of the 60S ribosomal subunit, preventing binding of elongation factor to the ribosome and thus inhibiting protein synthesis, resulting in cell death by apoptosis (Bergan et al., 2012).

The genes for Stx in *E. coli* are exclusively located on temperate lysogenic phages that integrate into the genome of their host bacterium. In the lysogenic state, Stx genes are replicated as an integral part of the bacterial genome. Expression of the phage genes occurs when the phage lytic cycle is activated by induction of the SOS response. This triggers both the packaging of the genes encoding Stx to be into phage particles, which are assembled and released through lysis of the cell, and simultaneous production of the Stx protein. The released Stx-encoding bacteriophages have the potential to transduce other *E. coli* leading to dissemination of this virulence phenotype.

The SOS response, a high-activity repair response to damage of chromosomal DNA, is regulated by the interplay of the two major proteins RecA, an ATP-dependent protein with DNA-binding abilities, and LexA, the key repressor of SOS-induced genes. Activation of RecA in response to DNA damage mediates auto-cleavage of both LexA and prophage repressors, leading to bacteriophage and Stx production.

The importance of Stx for EHEC pathogenesis has driven efforts to develop novel compounds that interfere with this potent toxin. In this work we designed a high throughput screen (HTS) to identify compounds that preferentially affect expression of *stx2.* We focused on Stx2 because studies in primates have shown that administration of Stx2 alone can produce the symptoms of HUS, while administration of Stx1 at the same dose does not. In addition, epidemiological and *in vitro* studies demonstrate that Stx2a is more likely to be associated with more serious human disease (Persson et al., 2007; Manning et al., 2008; Fuller et al., 2011). For the most effective lead compound, we then modified key functional groups to reveal the components required for activity. Based on our phenotypic studies, we hypothesized that the inhibition of Stx expression might be due to inhibition of the SOS response protein RecA, which we tested by a series of biochemical and biophysical analyses. Our work demonstrates that RecA is highly susceptible to inhibition and that targeting this protein is a viable approach to limiting production of Stx2 by EHEC.

## Materials and Methods

### Chemical Synthesis

Tetrahydrofuran and dichloromethane were purified through a Pure Solv 400-5MD solvent purification system (Innovative Technology, Inc). Solvents were evaporated under reduced pressure at 40°C. All reactions described were performed under argon atmosphere unless otherwise stated and monitored by thin layer chromatography (TLC) with pre-coated TLC plates (Merck Silica Gel 60 F254). Plates were visualized by UV light (254 nm), iodine vapors or stained with anisaldehyde.

Details of the chemical synthesis are provided in Supplementary Methods and an overview in Supplementary Figure S1. Compounds were purified via flash column chromatography using silica gel (FlurochemSilica LC 60A) as the stationary phase. ^1^H NMR and ^13^C NMR spectra were recorded at 400 and 100 MHz or at 500 and 125 MHz using either a Bruker DPX Avance400 instrument or a Bruker Avancell500 instrument, respectively. IR spectra were obtained using a Shimadzu FTIR-8400 spectrometer.

High-resolution mass spectra were obtained using a JEOL JMS-700 mass spectrometer by electrospray and chemical ionization operating at resolution of 15,000 full width at half height.

### High Throughput Screens

In preparation for screening experiments, plasmids pNY16, pNY14, and pAJR145 were transformed into ZAP0273 (*E. coli* Sakai *stx*-). These plasmids comprise the promoter region of the gene encoding *stx2, sulA* and *rpsM*, respectively fused to green fluorescent protein (GFP). The ChemBridge library of 17,500 chemically diverse compounds was screened with the pNY16, pNY14, and pAJR145 reporter plasmids at 50 μM and 1% DMSO. Gain settings for the fluorescence readings were optimized according to the zero percent effect (ZPE) values from each assay. The library is organized into 56, 384-well plates. The library was screened once with each assay and the *Z*′-values were analyzed for each plate. Fluorescence levels were measured at both *t* = 0 and at the assay end-points. Hits were defined as compounds that affected the fluorescence signal by greater than 3 SDs.

### Reporter Assays

Shiga toxin expression assays were performed using the p*stx2::GFP* reporter fusion plasmid (pNY16). The plasmid was transformed by electroporation into ZAP0273 and transformants cultured overnight in LB medium containing 35 μg/mL chloramphenicol. Overnight cultures were diluted into fresh LB at an OD_600_ of 0.08, and grown to an OD_600_ of 0.6 at 37°C and 200 rpm. Compounds and mitomycin C (MMC, 1 μg/mL) were added at this point, and fluorescence and optical density of cultures measured hourly. GFP fluorescence of 200 μL aliquots was measured in a 96-well blank microtiter plate using a FLUOstar Optima plate reader (BMG, Germany).

### Induction and Measurement of Stx Production in EHEC

Cultures of strain ZAP1620, a phage-type (PT) 21/28 EHEC isolate, were cultured overnight in LB medium. After dilution 1/100, cultures were grown in the presence or absence of AHU3 (50 μM) to exponential phase (OD_600_ = 0.3–0.4) before phage lysis was induced by the addition of mitomycin C (5 μg/ml). Lysis was allowed to proceed for 1.5 h after which cultures were filtered (0.22 μm filter). Supernatant samples containing the toxin were applied directly to a RIDASCREEN^®^ verotoxin (R-Biopharm) 96-well microtitre plate and assayed for toxin levels by ELISA according to manufacturer guidelines.

### Phage Transduction

Prophage strains *E. coli* JP10819 and *S. aureus* JP5011 (Quiles-Puchalt et al., 2014) were grown in LB (*E. coli*) or TSB (*S. aureus*) media at 37°C, and 50 μM AHU3 and 2 μg/ml MMC added at OD_600_ = 0.25. Cultures were then incubated at 32°C with 80 rpm shaking for 4 h, followed by 21°C without shaking for 16 h. Cultures were filtered through a 0.2 μM filter, and serially diluted in phage buffer (100 mM NaCl, 0.5 M tris pH 7.8, 1 mM MgSO_4_, 4 mM CaCl_2_). Hundred microliter of phage lysate was added to 1 ml aliquots of MG1655 (*E. coli*) or RN4220 (*S. aureus*) at an OD_600_ of 1.4. Samples were incubated without shaking at 37°C for 30 min before addition of 3 ml 0.75% agar and plating onto LB or TSB agar plates containing 6 μg/ml tetracycline and 1.7 mM sodium citrate. A negative control lacking addition of phage lysate was also included to confirm the absence of phage contamination. Colonies were subsequently counted to define the number of transduction events per sample.

### Colorimetric ATPase Assay

Colorimetric ATPase assays were performed using an Innova Biosciences ATPase assay kit (#601-0120), and reactions carried out following the manufacturer’s protocol, using 50 μM RecA (NEB), 500 μM poly dT ssDNA (Midland Certified Reagent Company) and 0.1-100 μM AHU3.

### Sedimentation Equilibrium (SE) Analytical Ultracentrifugation (AUC)

Analytical ultracentrifugation (AUC) was performed using an Optima XL-I analytical ultracentrifuge (Beckman Coulter), with samples in 50 mM Na-citrate, 5% (w/v) glycerol, pH 6.0, buffer conditions optimal for maximizing the oligomeric state of RecA (Brenner et al., 1990). Rotor speeds of 6, 10, and 14 k rpm at a temperature of 4 and 20°C were used, and absorbance scans (280 nm) were taken every 3 h until analysis of the scans with WinMATCH (Jeffrey Lary, University of Connecticut, Storrs, CT, USA) indicated that equilibrium had been reached. Sedimentation equilibrium data were analyzed with SEDPHAT (Vistica et al., 2004) using the single species analysis model in order to gain a model-independent measure of the whole-cell weight average molecular mass.

## Results

### Generation of Reporter Plasmids to Facilitate HTS

The main aim of our study was to identify compounds that specifically reduced Stx2 production by ZAP0273. To address this, we designed a HTS for compounds that preferentially affected expression of *stx2*, we also included a screen for *sulA* expression to examine generic effects on SOS induction (Mizusawa et al., 1983). The promoter regions of *stx2* and *sulA* were amplified from *E. coli* O157:H7 Sakai by PCR and cloned into the XbaI site upstream of the GFP gene in plasmid pKC26, thus creating transcriptional GFP reporter plasmids pNY16 and pNY14. A further control, pAJR145, was also used, comprising the promoter for *rpsM*, encoding the 30S ribosomal protein S13, fused to GFP. Expression of this plasmid is closely linked to bacterial growth rate. The use of GFP reporters allowed us to use intact bacteria in the screen, which ensures that any active compounds are likely to penetrate cells and reach intracellular targets.

### HTS for Compounds that Reduce Stx Expression

**FIGURE 1:**
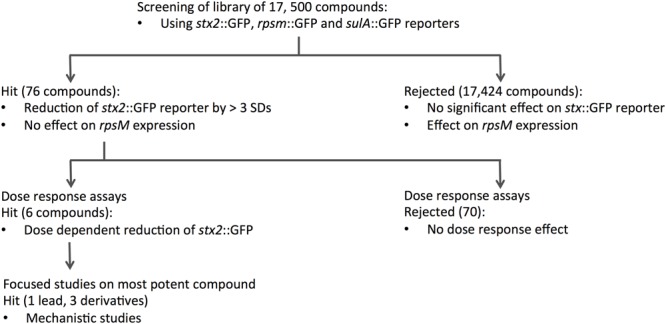
**Flow chart summarizing the screening process and DR experiments.** A library of 17,500 compounds was screened using *stx2::*GFP, *rpsm*::GFP and *sulA*::GFP reporter fusions. Seventy-six compounds that showed significant activity on *stx2::*GFP and *suIA*::GFP were identified from this initial screen, and further dose-response assays narrowed this to six hits that reduced *stx2*::GFP expression in a dose-dependent manner. The single most potent compound was taken forward for mechanistic studies.

To identify new compounds with the potential to reduce expression of Stx we performed a HTS of the Chem-Bridge library at the Laboratory for Chemical Biology, Umeå. The library comprises 17,500 chemically diverse compounds that have previously proven valuable for the identification of novel therapeutics. Each compound was initially tested at a concentration of 50 μM for the ability to suppress MMC-induced expression of *stx2*::GFP in ZAP0273. Further details of the HTS are provided in the Section “Materials and Methods” and **Figure 1**. The primary selection criteria was for drugs that inhibited pNY16 (*stx2*::GFP) fluorescence induction but which had limited effects on pAJR145 (*rpsm*::GFP) and pBY14 (*sulA*::GFP) expression. However, compounds that showed strong suppression of *stx2*::GFP expression and some suppression of *sulA*::GFP were also taken forward for further investigation. In total 76 hits, comprising 48 compounds that reduced fluorescence in the pNY16 screen only and 28 compounds that reduced fluorescence in pNY16 and pNY14 screens, were selected.

**FIGURE 2:**
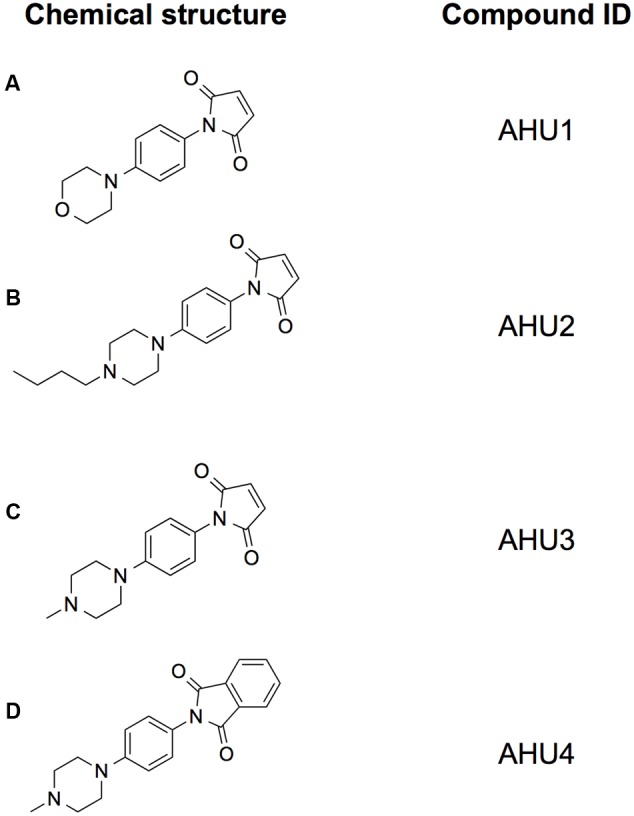
**Structures of the compounds used in this study.** Chemical structures of the hit compound AHU1 **(A)**, and its derivatives AHU2 **(B)**, AHU3 **(C)**, and AHU4 **(D)**. The maleimide moiety was determined to be essential for the molecule to be biologically active since its loss in the derivative AHU4 led to inactivity.

**FIGURE 3:**
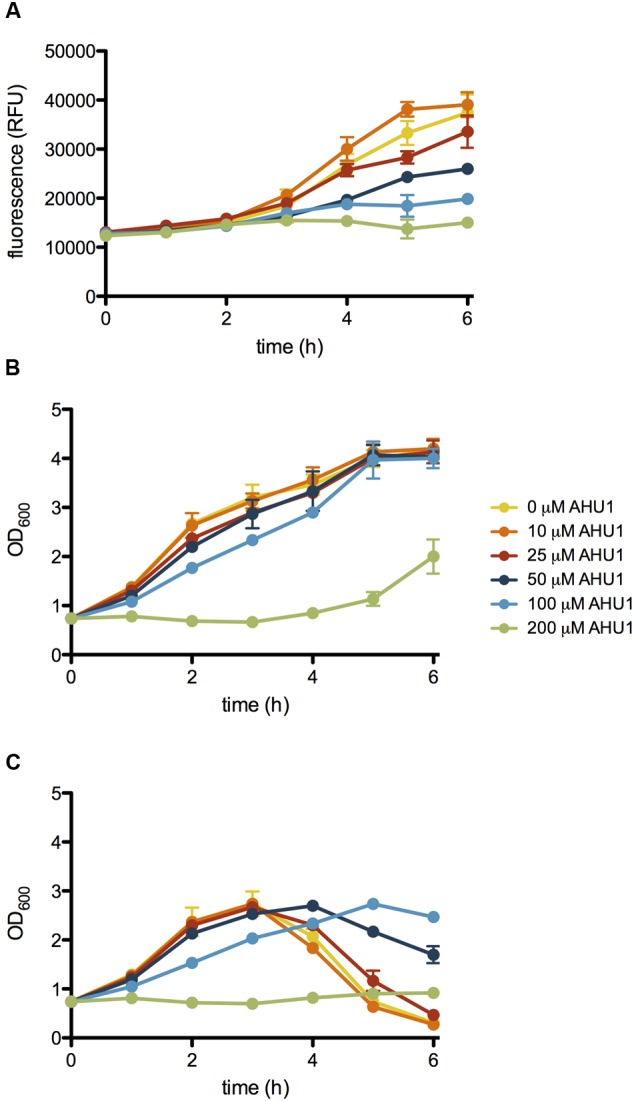
**Effect of AHU1 on *stx2*::GFP expression, growth rate and lysis of *E. coli*.** ZAP0273 transformed with pNY16 was cultured in the presence of 25-200 μM AHU1, and induced with 1 μg/ml MMC. Expression of *stx2*::GFP expression was measured by fluorescence, and bacterial growth by OD_600_. **(A)** Inhibition of MMC-induced *stx2*::GFP expression by AHU1. **(B)** Effect of AHU1 on bacterial growth in the absence of MMC. **(C)** Inhibition of MMC-induced bacterial lysis by AHU1. Experiments were performed in triplicate, and data plotted as the mean with standard deviation from the mean displayed by error bars.

To validate the candidates and eliminate false positives, compounds from the primary screens were studied in dose-response (DR) experiments. Concentrations of compounds ranging from 0.2 to 200 μM were prepared using 2-fold serial dilutions. The DR experiments were performed with the pNY16, pNY14, and pAJR145 reporters to evaluate inhibitor potency and specificity. From the initial screen of 76 primary hits, 6 compounds showed a reproducible dose-dependent reduction of MMC-induced *stx*::GFP expression (Supplementary Table S1). The most effective of these was Hit2Lead compound ID 5324836 (**Figure 2A**), a phenyl pyrroledione henceforth called AHU1. This compound displays dose-dependent suppression of MMC-induced *stx2*::GFP expression (**Figure 3A**) with no effect on growth rate at concentrations up to 50 μM (**Figure 3B**). Increasing the AHU1 concentration to 100 μM further reduced *stx2* expression but also inhibited the growth rate by 10%. As the genes encoding Stx are encoded on lytic phages we also tested the ability of AHU1 to affect bacterial lysis caused by phage mobilization. Addition of MMC to ZAP0273 results in phage-mediated lysis that can be assayed by monitoring the optical density of the culture. Lysis is evident around 3 h after addition of MMC but is markedly reduced by AHU1 at concentrations of 50 μm or greater (**Figure 3C**).

### Generation of a Focused Sub-Set of Compounds to Study Structure-Activity Relationship

AHU1 is a planar molecule composed of a maleimide group and a morpholine moiety, connected by a benzene ring (**Figure 2A**). In order to acquire information about the structure-activity relationship of this compound, chemical derivatives of this compound were synthesized. The morpholine group was chosen for modification, as the presence of a heteroatom on the six-membered ring readily facilitates chemical manipulation. Briefly, the morpholine ring was replaced with its bioisostere piperazine and the addition of a methyl group and a butyl chain on this position gave the compounds AHU2 and AHU3, respectively (**Figures 2B,C**). As we considered the maleimide part of the compound was likely to be central to the activity of the molecule, compound AHU4 was produced by the addition of a phenyl group to the maleimide moiety (**Figure 2D**).

### Effect of AHU1-4 on *stx* Expression

**FIGURE 4:**
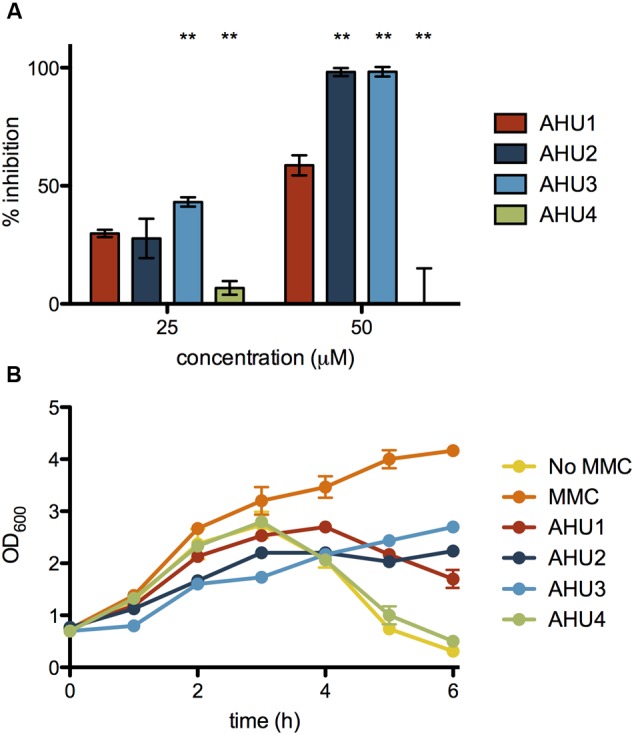
**Inhibition of *stx2*::GFP expression and MMC-induced lysis by AHU1-4. (A)** Inhibition of *stx2*::GFP expression by 25 and 50 μM AHU1-4 at 6 h after addition of AHU1-4 and MMC. Data were calculated from triplicate experiments and displayed as the mean inhibition with error bars showing the standard deviation from the mean. Asterisks indicate a significant difference (^∗∗^*p* < 0.001) from the original AHU1 concentration inhibition, determined by Student’s unpaired *t*-test. **(B)** Inhibition of MMC-induced bacterial lysis after addition of AHU1-4 and MMC. Experiments were performed in triplicate, and data plotted as the mean with standard deviation from the mean displayed by error bars.

To determine the activity of AHU1-4 we used the *stx2*::GFP reporter and monitored expression following addition of MMC. Experiments were performed over a range of concentrations (10-200 μM) for all four compounds. The effectiveness of AHU1-4 was directly compared by calculating the percentage inhibition of *stx2*::GFP expression 6 h post-MMC induction, at two concentrations of the compounds, 25 and 50 μM, respectively. Addition of AHU4, containing the phenyl substitution, resulted in no reduction in *stx*::GFP expression thereby demonstrating the importance of the maleimide group for activity. However, both AHU2 and AHU3 at 50 μM produced near-complete inhibition of *stx2*::GFP, which was significantly (*p* < 0.05) higher than AHU1, which when used at the same concentration inhibited expression by approximately 60% (**Figure 4A**). AHU3 produced a greater increase in inhibition at 25 μM when compared with AHU1. AHU2 and AHU3 also resulted in inhibition of phage-mediated bacterial lysis (**Figure 4B**). As AHU3 was the most effective inhibitor out of the four compounds, it was selected for use in all further experiments.

### Effect of AHU3 on Stx2 Production by EHEC

To assess if AHU3 could impact on Stx2 production in EHEC we directly assayed toxin production in ZAP1620, a wild-type strain that is lysogenized with both Stx2a- and Stx2c-encoding phages and was isolated from a human patient. ZAP1620 was cultured to mid-exponential phase and MMC added to induce toxin expression. Culture supernatants were isolated and the levels of toxin assessed using an ELISA-based assay (RIDASCREEN^®^ verotoxin, R-Biopharm). Data were calculated as a percentage of expression compared with the positive control (100%), consisting of inactivated Stx. Analysis of the supernatant of ZAP1620 showed strong MMC-dependent production of Stx2, at 85% (±7%) activity compared with the positive control. Addition of 50 μM AHU3 reduced Stx2 expression to 47% activity (±3%) compared with the positive control, demonstrating that AHU3 markedly inhibits Stx2 production in wild-type isolates.

### Effect of AHU3 on Phage Transduction

Having demonstrated that AHU3 was indeed a *bona fide* inhibitor of Stx expression, we focused our attention on understanding the mechanism of action underpinning its activity. As AHU3 was able to reduce phage-induced lysis of EHEC, we hypothesized that it might be able to reduce the generation of transducible phage particles. Phage transduction assays were performed using *E. coli* JP10819 that carries only the lysogenic Stx2 prophage ϕP27. This prophage contains a tetracycline resistance cassette inserted into the *stx2* toxin gene, and lacks other lysogenic prophages. Phage production was induced by addition of MMC in the presence and absence of 50 μM AHU3 and the resultant phages produced were isolated by filtration. The non-lysogenic strain *E. coli* MG1655 was used as a recipient for the phage isolated from *E. coli* JP10819.

**FIGURE 5:**
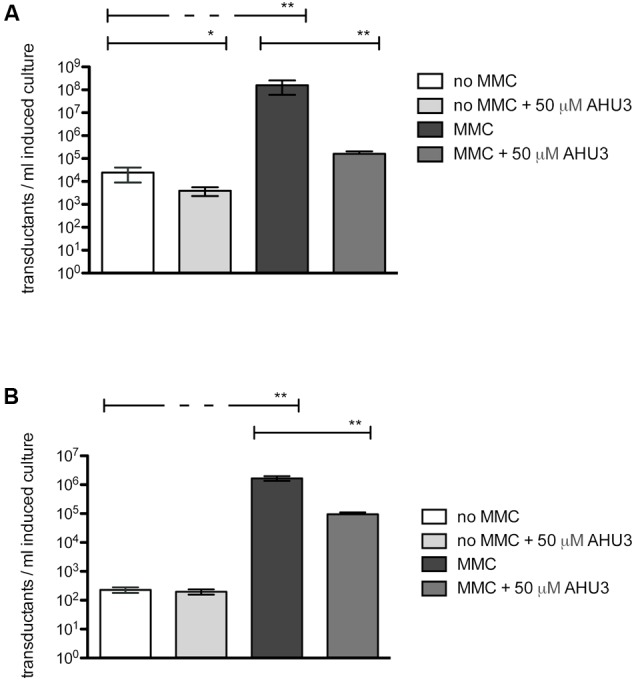
**Reduction of MMC-induced phage production by 50 μM AHU3. (A)** Induction of JP10819 with 2 μg/ml MMC resulted in significantly increased Stx phage production. Addition of 50 μM AHU3 produced a significant decrease in phage production by both non-induced and MMC-induced JP10819. The interaction between MMC and AHU3 was determined to be significant (*p* < 0.001), implying that the reduction in phage production by MMC-induced cells observed is not the result of the inhibitory effect of AHU3 on bacterial growth. **(B)** Induction of JP5011 with 2 μg/ml MMC resulted in significantly increased SLT phage production. Addition of 50 μM AHU3 produced a significant decrease in phage production by MMC-induced JP5011. Asterisks indicate a significant difference (^∗^*p* < 0.05, ^∗∗^*p* < 0.001) in phage production between the groups indicated by capped lines. The data shown are the average of triplicate individual experiments with standard deviation from the mean displayed as error bars. Statistical significance was determined by GLM analysis.

When no MMC was added to *E. coli* JP10819, basal levels of Stx phage (10^4^ phage/ml) were observed which increased by four logs to 10^8^ phage/ml following induction with MMC (**Figure 5A**). The presence of 50 μM AHU3 resulted in a significantly (*P* < 0.001) lower amount of phage produced with a titre of 10^5^ phage/ml; demonstrating that the production of functional Stx phage particles is inhibited by AHU3.

As the AHU compounds appeared to inhibit phage-mediated bacterial lysis, we next explored whether these drugs could inhibit expression of other prophages. To evaluate their activity in Gram-positive bacteria, *Staphylococcus aureus* JP5011, which carries the ϕSLT phage containing a tetracycline resistance cassette inserted into the *plv* gene, was used. When induced by 2 μg/ml MMC, approximately 10^6^ transductants/ml of JP5011 culture were produced. In contrast, only 10^2^ transductants/ml were generated from uninduced JP5011. Treatment of the MMC-induced culture with 50 μM AHU3 showed greater than a log fold reduction in the number of transductants generated (**Figure 5B**).

### Effect of AHU3 on RecA Expression and Activity

Given that AHU3 inhibits expression and formation of functional phage particles, we hypothesized that the compound may function by affecting the activity of a protein involved in the SOS response required for induction of phage expression. When the SOS response is initiated, RecA binds to single stranded DNA, forming filaments with ATPase activity. These activated RecA filaments mediate autocleavage of the key SOS response repressor LexA and prophage repressors, allowing gene expression and production of assembled phage particles. Therefore, one candidate target protein was RecA, the activity of which can be measured using assays that quantify the amount of free phosphate as a measure of ATP hydrolysis. This provides an indirect measure of RecA nucleoprotein filament assembly (Wigle and Singleton, 2007; Wigle et al., 2009; Sexton et al., 2010).

**FIGURE 6:**
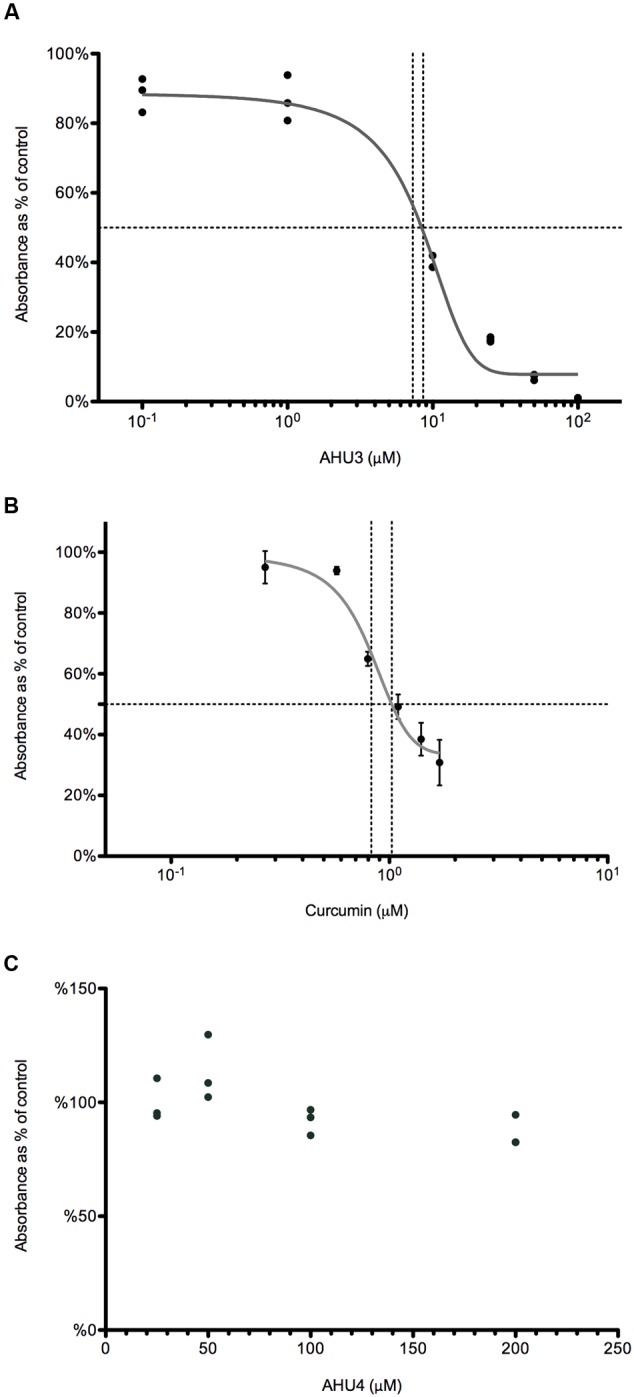
**AHU3 inhibits RecA-mediated ATP hydrolysis. (A)** Dose-response curve for AHU3 showing the inhibition of ATPase activity with 0.1-100 μM AHU3. Data are plotted as a percentage of the average absorbance of the assay wells containing 0 μM AHU3. The curve exhibits an IC_50_ value of 7.72 μM and a Hill slope of 0.109. **(B)** Dose-response curve for curcumin showing the inhibition of ATPase activity with 0.1-100 μM curcumin. The curve exhibits an IC_50_ value of 6.69 μM and a Hill slope of 2.400. **(C)** Dose-response curve for AHU4 showing no inhibition of ATPase activity.

Using an ATPase assay (Innova Biosciences) we optimized RecA and ssDNA concentrations at 250 nM and 5 μM, respectively. DMSO, required for solubility of AHU3, was kept at a final concentration (2%), which did not interfere with the enzymatic activity of RecA. The ability of AHU3 to inhibit RecA-mediated ATP hydrolysis was investigated at concentrations between 0.1 and 100 μM, revealing a concentration-dependent decrease in enzymatic activity. The data were used to generate a dose-response curve (**Figure 6A**) which was used to determine a relative IC_50_ value of 7.72 μM using GraphPad Prism. The known RecA inhibitor curcumin (15) was used as a positive control (**Figure 6B**) giving a relative IC_50_ of 6.69 μM. Since the AHU4 compound showed no reduction in *stx*::GFP expression, it was used as the negative control (**Figure 6C**). As expected, AHU4 showed no dose-dependent effect on RecA-mediated ATP hydrolysis

### Effect of AHU3 on RecA oligomerization

**FIGURE 7:**
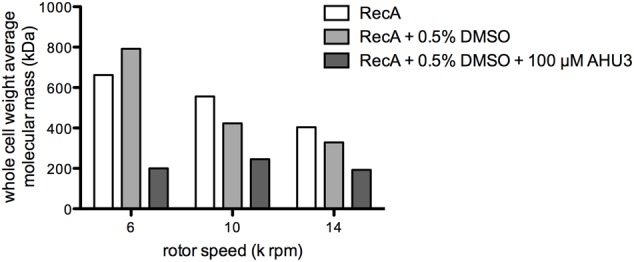
**AHU3 decreases RecA oligomerization.** Whole cell weight average molecular mass of RecA at AUC rotor speeds of 6, 10, and 14 k rpm. Ten micrometer RecA was studied alone, in the presence of 0.5% DMSO, and in the presence of 0.5% DMSO and 100 μM AHU3.

Analytical ultracentrifugation allows the quantitative analysis of macromolecules in solution. Previous studies have used AUC sedimentation equilibrium experiments (SE) to analyze the oligomerization of *E. coli* RecA, revealing monomers in reversible equilibrium with trimers, hexamers and dodecamers (12). To assess whether AHU3 affects the oligomerization dynamics of the protein, we performed SE for RecA in the presence and absence of AHU3. We also controlled for any influence of DMSO, included as a solvent for AHU3, on protein behavior. The SE data were analyzed with SEDPHAT using the species analysis model with a single species, in order to gain a model-independent measure of the whole-cell weight average molecular mass. The average molecular mass of RecA oligomers in the absence of AHU3 and DMSO was 556 kDa at 10,000 rpm and 404 kDa at 14,000 rpm (**Figure 7**). When AHU3 was present in the sample, the average molecular mass of RecA oligomers was greatly reduced at all rotor speeds tested. The greatest changes were observed at 6,000 rpm, with a 70% decrease in the average molecular mass compared with untreated RecA (**Figure 7**). At 10,000 and 14,000 rpm, the decreases were still marked, at 56 and 52%, respectively. As a control we also compared the average molecular mass of RecA with that of the same protein in the presence of DMSO and found only very minor effects on RecA oligomerization. These data demonstrate that AHU3 is affecting the formation of larger oligomeric RecA species, resulting in an increase in lower molecular mass oligomers.

## Discussion

In this study we employed a HTS to identify compounds with an inhibitory effect on *stx2* expression. We used bacterial GFP reporter assays to ensure that any hits were active against intact bacteria. Our selection criteria included a marked reduction in *stx2* expression, with minimal effects on growth rate or off-target genes. When coupled with studies to determine the dose-dependency of the response, only 6 compounds met our criteria and we selected the best performing of these, AHU1, for further study. Structure-activity studies resulted in the synthesis of AHU3 that showed inhibition of *stx2* gene expression and phage production at a concentration that had minimal impact on bacterial growth. Moreover, AHU3 was shown to be a genuine inhibitor of Stx2 production and reduced toxin expression in a wild-type EHEC O157 strain. However, the ability of AHU3 to inhibit bacteriophage-mediated lysis, along with its activity in both Gram-negative and Gram-positive strains indicated that the activity of this compound was specific to an essential component of the SOS response, rather than specifically on Stx production.

Initiation of the SOS response is controlled by RecA, the activity of which requires formation of ssDNA-bound helical homopolymeric filaments (Chen et al., 2008). We therefore hypothesized that RecA was the biological target of AHU3, as inhibition of either activation of the monomeric form of this protein or function of assembled RecA filaments would prevent autocleavage of the repressors of SOS and phage genes.

The substitution of the AHU3 maleimide group with a phenyl group, generating AHU4, caused the compound to become inactive. Although natural maleimides are rare in nature, several small compounds containing a modified maleimide moiety have been identified in fungal and bacterial organisms. Pencolide, produced by Penicillium strains, exhibits both bacteriostatic and fungicidal activity (Lucas et al., 2007), while the fungal compound farinomalein shows potent activity against plant pathogen oomycetes (Putri et al., 2014). A *Streptomycin showdoensis* maleimide compound, showdomycin, targets oxidoreductases, and transferases involved in major cellular functions associated with virulence, growth, and persistance in both Gram-negative and Gram-positive strains (Böttcher and Sieber, 2010).

Maleimide moieties are well known as Michael acceptors, and react readily with sulfhydryl groups of cysteine residues. It is therefore highly likely that AHU3 reacts with RecA through this addition-elimination mechanism, covalently binding to one or more of the three cysteine residues present in RecA. This mechanism of inhibition has been reported for other small maleimide-based molecules; a phenyl-substituted maleimide with anti-angiogenic activity shows potential as a candidate for treatment of proliferative retinal disorders (Nowak-Sliwinska et al., 2012), while a series of maleimide-based compounds with inhibitory activity on monoamine oxidase B have shown promise as a potential therapy for Parkinson’s disease (Manley-King et al., 2009). Interestingly, a chloromaleimide-based compound bearing a striking structural resemblance to AHU3 has been reported as an inhibitor of RAD51, the human homologue of RecA. This compound, RI-1, was shown to covalently bind a single cysteine residue on a RAD51 located in the interface required for monomer-monomer interaction (Budke et al., 2012). In support of this hypothesis, we showed that AHU3 directly affected RecA in solution by reducing the formation of larger oligomeric species. The effects of these compounds on eukaryotic cells will form part of ongoing studies into the possible application of these molecules to treat infection-based pathology in relation of phage-induced toxins. However, this is dependent on lack of cross reactivity for RAD51.

Several groups have suggested RecA as an attractive target for inhibition and performed specific screens to identify suitable compounds (Wigle and Singleton, 2007; Wigle et al., 2009; Sexton et al., 2010; Peterson et al., 2012). Bacterial strains with RecA deletion or inactivation have displayed an increased susceptibility to a number of antimicrobials and a delayed emergence of resistance compared with wild-type strains (Singh et al., 2010; Thi et al., 2011; Alam et al., 2016). Additionally, the production and release of bacteriophages initiated by the SOS response can result in horizontal transmission of genes located on mobile genetic elements, and therefore inhibition of this process may limit the dissemination of virulence factors and antibiotic resistance (Beaber et al., 2004).

While RecA inhibitors might not show a high degree of selectivity due to the high conservation of RecA across bacteria, they may have potential as drugs administered alongside other more specific treatments to increase bacterial susceptibility to anti-bacterials or limit development of antibiotic resistance. Our work has demonstrated that RecA inhibition can also significantly limit expression of prophage genes encoding virulence factors, including Stx, emphasizing their additional potential as therapies to limit bacterial production of key phage-encoded virulence factors.

## Author Contributions

AH-U and ZM contributed equally to this work and performed the chemical synthesis and analysis of the mechanism of action. NY and HU performed the HTS. SF performed all work associated with clinical isolates. NC and JC performed the phage transduction assays. GD, ME, OB, RM, DG, and AR conceived and supervised various aspects of the study. All authors have read, contributed and approved the manuscript.

## Conflict of Interest Statement

The authors declare that the research was conducted in the absence of any commercial or financial relationships that could be construed as a potential conflict of interest.
