# Supplementary material for: Identification and Characterization of Novel Compounds Blocking Shiga Toxin Expression in Escherichia coli O157:H7
Source: Front Microbiol. 2016 Nov 30;7:1930. doi: 10.3389/fmicb.2016.01930 (PMC5127787; doi:10.3389/fmicb.2016.01930)
Supplement: Supplementary file 1 [file Data_Sheet_1.pdf]

Supplementary Figure 1: Synthesis of N-arylmaleimides.

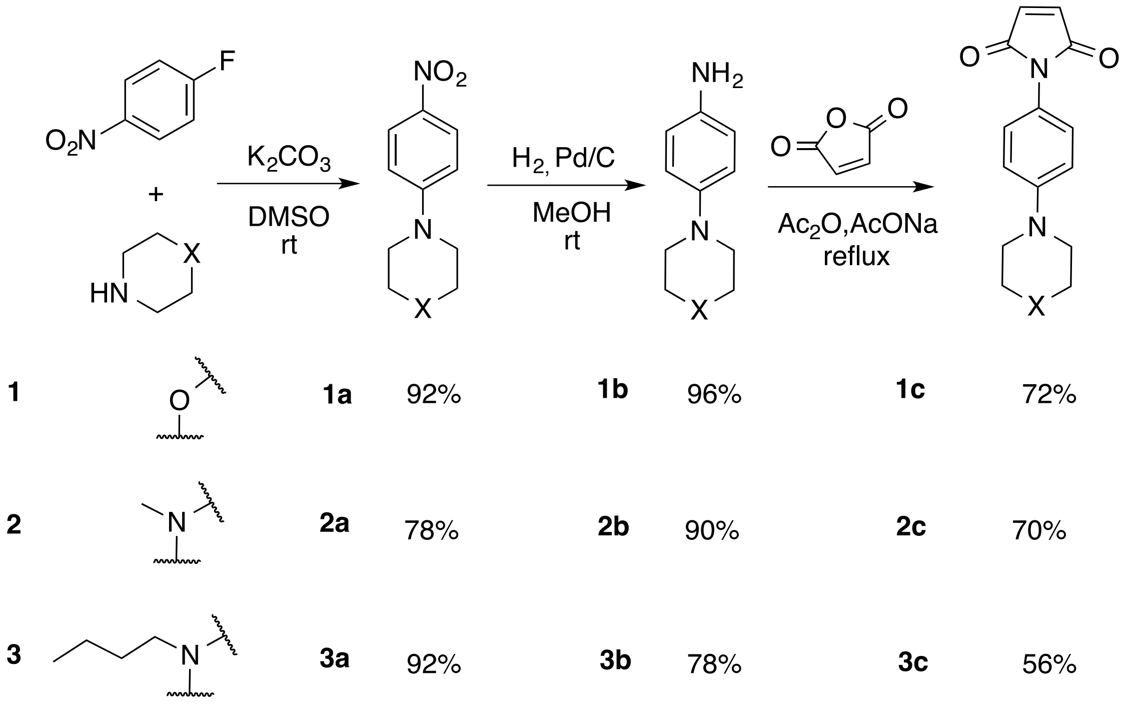

4-(4-nitrophenyl)morpholine (1a). 1-fluoro-4-nitrobenzene (0.5 g, 3.5 mmol) and K_2CO_3 (0.53 g, 3.8 mmol) were stirred in DMSO (0.75 mL) for 0.5 h at room temperature. Then, morpholine (0.30 mL, 3.5 mmol) was added slowly to the reaction mixture and this was left stirring overnight. Subsequently, the contents were poured into ice-water and the precipitate was filtered and dried to afford **1a** (0.6 g, 92%) as a yellow powder. The NMR data obtained is in accordance with the literature.

1-methyl-4-(4-nitrophenyl)piperazine (1b). Following the procedure for compound **1a**, 1-fluoro-4-nitrobenzene (0.5 g, 3.5 mmol), K_2CO_3 (0.53 g, 3.8 mmol), DMSO (0.75 mL) and 1-methylpiperazine (0.39 mL, 3.5 mmol) afforded **1b** (0.72 g, 92%) as a yellow powder. The NMR data obtained is in accordance with the literature. ^{1}H NMR ($CDCl_3$, 400 MHz) δ: 8.14 (2H, d, *J*=9.5 Hz), 6.85 (2H, d, *J*=9.5 Hz), 3.46 (4H, m), 2.58 (4H, m), 2.38 (3H, s).

1-buthyl-4-(4-nitrophenyl)piperazine (1c). Following the procedure for compound **1a** 1-fluoro-4-nitrobenzene (0.5 g, 3.5 mmol), K_2CO_3 (0.53 g, 3.8 mmol), DMSO (0.75 mL) and 1-butylpiperazine (0.5 mL, 3.5 mmol) afforded **1c** (0.72 g, 78%) as a yellow powder. The NMR data obtained is in accordance with the literature. ^{1}H NMR ($CDCl_3$, 400 MHz) δ: 8.15 (2H, d, *J*=9.5 Hz), 6.85 (2H, d, *J*=9.5 Hz), 3.47 (4H, m), 2.61 (4H, m), 2.43 (2H, m), 1.54 (2H, m), 1.35 (2H, sextet, 7.2 Hz), 0.97 (3H, t, 7.3 Hz).

4-morpholinoaniline (1b). To a mixture of 4-(4-nitrophenyl)morpholine (0.5 g, 2.4 mmol) in anhydrous methanol (7 mL) under atmosphere of argon was

added Pd/C (50 mg, 10%) followed by replacement of the argon with a hydrogen atmosphere. The reaction mixture was stirred at room temperature until completion (2 h). The crude mixture was filtered over celite and the methanol was concentrated in vacuo to afford **1b** (0.41 g, 96%) as a beige powder. The NMR data obtained is in accordance with the literature. ^{1}H NMR ($CDCl_3$, 400 MHz) δ: 6.83 (2H, d, *J*=8.8 Hz), 6.70 (2H, d, *J*=8.8 Hz), 3.88 (4H, m), 3.05 (4H, m).

4-(4-methylpiperazin-1-yl)aniline (2b). Following the procedure for compound **2a**, 1-methyl-4-(4-nitrophenyl)piperazine (0.7 g, 3.1 mmol), anhydrous methanol (15 mL) and Pd/C (70 mg, 10%) afforded **2b** (0.47 g, 78%) as a brown powder. The NMR data obtained is in accordance with the literature. ^{1}H NMR ($CDCl_3$, 400 MHz) δ: 6.85 (2H, d, *J*=8.9 Hz), 6.69 (2H, d, *J*=8.9 Hz), 3.10 (4H, m), 2.61 (4H, m), 2.38 (3H, s).

4-(4-butylpiperazin-1-yl)aniline (3b). Following the procedure for compound **2a**, 1-butyl-4-(4-nitrophenyl)piperazine (0.7 g, 2.7 mmol), anhydrous methanol (15 mL) and Pd/C (70 mg, 10%) afforded **3b** (0.63 g, 90%) as a brown powder. The NMR data obtained is in accordance with the literature. ^{1}H NMR ($CDCl_3$, 400 MHz) δ: 6.84 (2H, d, *J*=8.9 Hz), 6.67 (2H, d, J=8.9 Hz), 3.09 (4H, m), 2.63 (4H, m), 2.41 (2H, m) 1.53 (2H, m) 1.37 (2H, sextet, *J*=7.2 Hz) 0.95 (3H, t, *J*= 7.3 Hz).

1-(4-morpholinophenyl)-1*H*-pyrrole-2,5-dione (1c). A solution of 4-morphoaniline (0.2 g, 1.12 mmol) in DCM (5 mL) was treated by the slow

addition of maleic anhydride (0.1 g, 1.12 mmol). The reaction was stirred at room temperature for 1 h. Then, the DCM was evaporated in vacuo to afford the maleanilic acid, which was dissolved in acetic anhydride (5 mL) and sodium acetate (22 mg, 10%). The mixture was heated for 2 h under reflux. Then the reaction was cooled down, quenched with water and neutralised with a K_2CO_3 saturated solution. The solution was then transferred to a separation funnel and extracted with DCM. The combined organic layers were dried with Na_2SO_4 and concentrated in vacuo. The product was purified through column chromatography (petroleum ether/AcOEt, 5/5) giving 0.20 g (72%) of **1c** as an orange powder. ^1H NMR ($CDCl_3$, 400 MHz) δ : 7.13 (2H, d, J=9.1 Hz), 6.89 (2H, d, 9.1 Hz), 6.75 (2H, s), 3.79 (4H, m), 3.11 (4H, m). 13C NMR (CDCl3, 100 MHz) δ : 169.9, 150.9, 134.1, 127.1, 122.8, 115.7, 66.8, 48.9. IR $υ_{max}$ (film)/cm^{-1} 30071, 2358, 1707, 1517. MS (EI) $[M]^{=}$= 258.2

1-(4-(4-methylpiperazin-1-yl)phenyl)-1*H*-pyrrole-2,5-dione (2c). Following the procedure for compound **1c**, 4-(4-methylpiperazin-1-yl)aniline (0.2 g, 1.04 mmol), maleic anhydride (0.1 g, 1.12 mmol), DCM (5 mL), acetic anhydride (5 mL) and sodium acetate (22 mg, 10%) yielded 0.13 g (56.2%) as an orange solid. ^1H NMR ($CDCl_3$, 400 MHz) δ : 7.22 (2H, d, *J*=9.1 Hz), 7.01 (2H, d, *J*=9.1 Hz), 6.86 (2H, s), 3.28 (4H, m), 2.61 (4H, m), 2.39 (4H, m). ^{13}C NMR ($CDCl_3$, 100 MHz) δ : 169.9 150.9, 134.1, 127.1, 122.3, 116, 55, 48.7, 46.1. IR v_{max} (film)/cm^{-1} 3089, 2947, 2804, 2359, 1700, 1517. MS (EI) $[M]^{=}$= 271.2

1-(4-(4-butylpiperazin-1-yl)phenyl)-1*H*-pyrrole-2,5-dione (3c). Following the procedure for compound **1c**, 4-(4-butylpiperazin-1-yl)aniline (0.2 g, 0.85

mmol), maleic anhydride (0.08, 0.85 mmol), DCM (5 mL), acetic anhydride (5 mL) and sodium acetate (22 mg, 10%) yielded 0.19 g (70%) as an orange solid ^{1}H NMR ($CDCl_3$, 400 MHz) δ : 7.22 (2H, d, J=9.1 Hz), 7.01 (2H, d, *J*=9.1 Hz), 6.86 (2H, s), 3.28 (4H, m), 2.61 (4H, m), 2.39 (4H, m). ^{13}C NMR ($CDCl_3$, 100 MHz) δ : 169.9, 150.9, 134.1, 127.1, 122.2, 116, 58.4, 53.1, 48.7, 29, 20.7, 14. IR υ_{max} (film)/cm^{-1} 3086, 2925, 2359, 1700, 1517. MS (EI) $[M]^{=}$= 313.3

2-(4-morpholinophenyl)isoindoline-1,3-dione. Following the procedure for compound **1c**, 4-morpholinoaniline (0.090 g, 0.5 mmol), maleic anhydride (0.07 g, 0.5 mmol), DCM (3 mL), acetic anhydride (3 ml) and sodium acetate (0.01 g, 10%) yielded 0.14 g (96.5%) as a yellow solid. ^{1}H NMR ($CDCl_3$, 400 MHz) δ : 7.96 (2H, dd, *J*= 5.5, 3 Hz), 7.80 (2H, dd, *J*= 5.4, 3 Hz), 7.34 (2H, m), 7.03 (2H, m), 3.90 (4H, m), 3.24 (4H, m). ^{13}C NMR ($CDCl_3$, 100 MHz) δ : 167.6, 150.9, 134.2, 131.9, 127.4, 123.6, 123.2, 115.78, 66.83, 49. IR υ_{max} (film)/cm^{-1} 2952, 2864, 2364, 1707, 1517.

Table 1

Chemical structure	Compound ID
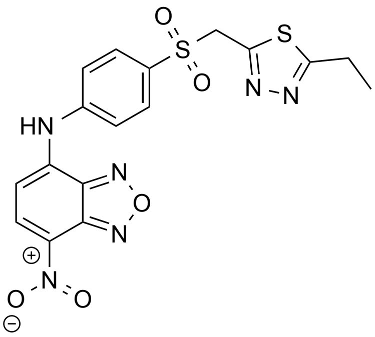	5241409
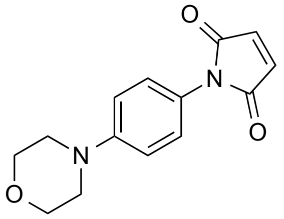	5324836
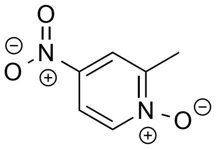	5140494
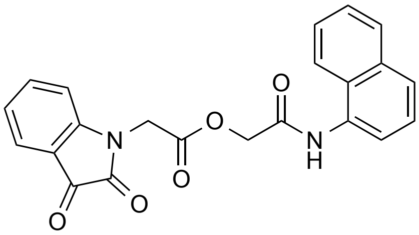	6343971
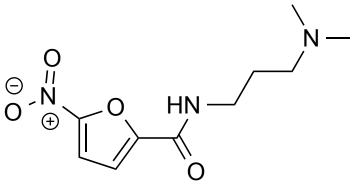	5528274
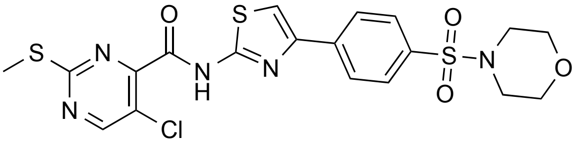	7988274
